# A candidate RxLR effector from *Plasmopara viticola* can elicit immune responses in *Nicotiana benthamiana*

**DOI:** 10.1186/s12870-017-1016-4

**Published:** 2017-04-14

**Authors:** Jiang Xiang, Xinlong Li, Ling Yin, Yunxiao Liu, Yali Zhang, Junjie Qu, Jiang Lu

**Affiliations:** 1 The Viticulture and Enology Program, College of Food Science and Nutritional Engineering, China Agricultural University, Beijing, China; 2 Center for Viticulture and Enology, School of Agriculture and Biology, Shanghai Jiao Tong University, Shanghai, 200240 China; 3 Guangxi Crop Genetic Improvement and Biotechnology Laboratory, Guangxi Academy of Agricultural Sciences, Nanning, China

**Keywords:** *Plasmopara viticola*, grapevine, RxLR effector, *Nicotiana benthamiana* cell death, immune responses

## Abstract

**Background:**

Diverse plant pathogens deliver effectors into plant cells to alter host processes. Oomycete pathogen encodes a large number of putative RxLR effectors which are likely to play a role in manipulating plant defense responses. The secretome of *Plasmopara viticola* (downy mildew of grapevine) contains at least 162 candidate RxLR effectors discovered in our recent studies, but their roles in infection and pathogenicity remain to be determined. Here, we characterize in depth one of the putative RxLR effectors, PvRxLR16, which has been reported to induce cell death in *Nicotiana benthamiana* in our previous study.

**Results:**

The nuclear localization, W/Y/L motifs, and a putative *N*-glycosylation site in C-terminal of PvRxLR16 were essential for cell death-inducing activity. Suppressor of G-two allele of Skp1 (SGT1), heat shock protein 90 (HSP90) and required for Mla12 resistance (RAR1), but not somatic embryogenesis receptor-like kinase (SERK3), were required for the cell death response triggered by PvRxLR16 in *N. benthamiana*. Some mitogen-activated protein kinases and transcription factors were also involved in the perception of PvRxLR16 by *N. benthamiana*. PvRxLR16 could also significantly enhance plant resistance to *Phytophthora capsici* and the nuclear localization was required for this ability. However, some other PvRxLR effectors could suppress defense responses and disease resistance induced by PvRxLR16, suggesting that it may not trigger host cell death or immune responses during physiological infection under natural conditions.

**Conclusion:**

These data demonstrate that PvRxLR16 may be recognized by endogenous proteins in nucleus to trigger immune responses in *N. benthamiana*, which in turn can be suppressed by other PvRxLR effectors.

**Electronic supplementary material:**

The online version of this article (doi:10.1186/s12870-017-1016-4) contains supplementary material, which is available to authorized users.

## Background

In nature, plants are attacked by various pathogens and protect themselves via sophisticated surveillance systems of innate immunity [1–3]. The first layer of the plant immune system is known as PAMP-triggered immunity (PTI) and has evolved to detect evolutionarily conserved molecular signatures of pathogens (pathogen- or microbe-associated molecular patterns (PAMPs/ MAMPs) and PRR-mediated immunity) [4, 5]. In turn, successful pathogens can secrete effector proteins into host cells to interfere with PTI, resulting in effector-triggered susceptibility (ETS). The second layer of the plant immune system is effector-triggered immunity (ETI), whereby plant resistance (R) proteins recognize pathogen effectors directly or indirectly, resulting in localized cell death known as the hypersensitive response (HR). These effector proteins recognized by *R* gene products of host plants are proposed to be avirulence (Avr) proteins [4, 6, 7].

Oomycetes are a major class of destructive plant pathogens, which contain over 800 species of downy mildew pathogens in 17 genera (*Peronosporaceae*), along with over 120 species in the *Phytophthora* genus [8, 9]. Oomycetes also deliver a wide diversity of effectors into plant cells to subvert host immunity as other pathogens [10]. To date, increasing numbers of *Avr* genes have been identified from different oomycetes, including *Phytophthora sojae*, *Phytophthora infestans* and *Hyaloperonospora arabidopsidis* [11–24]. Interestingly, all the Avr proteins from oomycetes, with one exception (ATR5), contain an N-terminal signal peptide which can direct the effector to the outside of the pathogen, followed by RxLR-EER motifs that are responsible for transporting effector into the interior of host cells [23, 25]. These effectors carrying RxLR motifs are defined as RxLR effectors which have been extensively studied in recent years [26]. The important discovery of RxLR effectors in oomycetes has expedited the identification process of avirulence genes [12].

Emerging evidences indicate that RxLR effectors target various subcellular compartments of plant cells to perform their functions. For instance, the effector Avh241 of *P. sojae* localizes to plasma membrane and the localization is required for its activity to induce cell death [27]. Functional analysis revealed the *H. arabidopsidis* effector HaRxL17 significantly enhances plant susceptibility which localizes to the tonoplast in uninfected cells and to membranes around haustoria in infected cells [28]. In another study, it was found that the effector AVRblb2 from *P. infestans* accumulates around haustoria and enhances susceptibility of hosts by preventing the host papain-like cysteine protease C14 secreting into the apoplast [29]. Similarly, the *P. infestans* RxLR effector AVR2 accumulates around haustoria with its host target BSL1, a putative phosphatase, to promote the association of BSL1 with R2, thereby triggering HR [30]. The plant cell nucleus is also considered one of the main targets for RxLR effector proteins. Many recent studies on interactions between plants and oomycete pathogens indicated that RxLR effectors target the host nucleus in order to modify host cell physiology. RxLR effector Pi04089 from the potato blight pathogen *P. infestans* localizes to host nucleus where it targets the putative potato K-homology (KH) RNA-binding protein, StKRBP1, to enhance colonization. Its nuclear localization is required for enhanced colonization of *P. infestans* [31]. Pi04314, another RxLR effector from *P. infestans*, also accumulates in the host nucleus and has the ability to impair induction of jasmonic and salicylic acid-responsive genes to enhance leaf colonization by *P. infestans* [32].

The hypersensitive response (HR)-like cell death is one of the most dramatic displays of programmed cell death (PCD) that is frequently observed in ETI [33]. Some host immune responses precede the HR in general, including proteolysis, changes in ion fluxes, accumulation of reactive oxygen species (ROS), increased levels of defense- related hormones, and activation of mitogen-activated protein kinase (MAPK) cascades [34]. The timely induction of HR in host plants seems to establish a formidable barrier to pathogen colonization, especially to biotrophic organisms which must absorb nutrients from living host cells. For example, ATR1, an avirulence protein of *H. arabidopsidis*, can elicit HR and a resistance response to pathogens in the presence of RPP1 [23]. Moreover, the avirulence effector AVR1 of *P. infestans* activates R1-mediated hypersensitive response and host defence when the AVR1/R1 pair is in the nucleus [35]. PAMPs may also elicit programmed cell death when recognized by pattern recognition receptors (PRRs) directly or indirectly. The PcINF1 elicitin from *P. capsici*, conceived as oomycete PAMPs, triggers cell death in pepper (*Capsicum annuum* L.), apparently by binding to a cell surface receptor SRC2–1 [36].

The obligate biotrophic oomycete *Plasmopara viticola* ([Berk. et Curt.] Berl. et de Toni) causes devastating downy mildew disease of grapevine all over the world. This pathogen needs to obtain all of its nutrition from living cells of grapevine to complete its whole life cycle [37]. However, the molecular basis of the interaction between *P. viticola* and the grapevine host is not well understood. Currently, a secretome of grapevine downy mildew was predicted by transcriptome sequencing analysis and then 51 RxLR effector candidates were identified [38]. Interestingly, one of these effectors, PvRxLR16, could directly trigger cell death in *N. benthamiana* which was highly expressed at the late infection stages (72 hpi). However, many other PvRxLR effectors fully blocked the programmed cell death elicited by PvRxLR16 [39]. Functional verification of this effector is of particular interest for the identifying potential virulence or avirulence factor.

In the present study, we aimed to further characterize the function of the effector protein PvRxLR16 in *N. benthamiana*. Sequence analysis revealed the presence of W/Y/L motifs and a putative *N*-glycosylation site. Deletions or mutations on these motifs abolished the cell death inducing activity of PvRxLR16. Some key proteins involved in signaling pathways of immunity were essential for the cell death response triggered by PvRxLR16. In addition to cell death, PvRxLR16 could elicit other basal defence responses, including ROS accumulation and defence-associated genes expression, resulting in disease resistance of *N. benthamiana* against *P. capsici*. However, the immune responses triggered by PvRxLR16 could be abolished successfully by other RxLR effectors from *P. viticola*.

## Methods

### Plant material and growth condition

Seeds of *Arabidopsis thaliana* (Col-0) purchased from The Arabidopsis Information Resource (TAIR) were cultivated in a phytochamber at 22 °C/16 h light and 20 °C/8 h dark cycle under 40%–60% humidity. *Nicotiana benthamiana* plants (provided by Prof. Guiqin Qu from China Agricultural University) were grown in a greenhouse at 25 °C with 16 h of illumination per day. Onion (*Allium cepa* L.) was purchased from a local supermarket.

### Plasmid construction

The primers used for the following plasmid constructs are shown in Supporting Information Additional file 1: Table S1. The various mutants of *PvRxLR16* for PVX assay were amplified using combinations of primers documented in Additional file 1: Table S1. The PCR products were cut with *Xma I* and *Sal I* restriction enzymes and ligated into the PVX::*flag* vector. For GFP fusion constructs, *PvRxLR16*, *PvRxLR16*:*NES* and *PvRxLR16:nes* were amplified with gene-specific primers modified to contain the Gateway (Invitrogen) attB recombinantion sites. The PCR amplicons were cloned into entry vector pDONR222 (Invitrogen) via BP reactions and subsequently recombined into the binary vector pH7FWG2, 0 using Gateway LR recombination. To make the constructs for virus induced gene silencing (VIGS) in *N. benthamiana*, partial sequences of purpose genes were amplified from cDNA of *N. benthamiana* and subsequently ligated into pTRV2 vector using the *Xma I* and *Kpn I* restriction sites. All the generated plasmids were validated by sequencing by Majorbio, Inc. (Shanghai, China). The schematic diagrams of constructs used in this study were shown in Additional file 2: Figure S1.

### Agroinfiltration and infection assays

Agroinfiltration assays in *N. benthamiana* were performed as described in our previous study [39]. Infection assays were performed by droplet inoculation of zoospore suspensions (100 of zoospores/μL) on detached *N.benthamiana* leaves. Zoospores were prepared as reported previously [40]. *Agrobacteria* (provided by Prof. Daolong Dou, Nanjing Agricultural University) cells containing plasmid constructs were infiltrated in *N.benthamiana* leaves. After 24 h, each leave was detached and inoculated with zoospore suspensions on the abaxial surface. The diameters of the infected areas were measured at 36 and 48 hpi, and photographed at 36 h under UV light. Data of at least three biological replicates were combined.

### Protoplast preparation of *A. thaliana* and transfection

The isolation of *A. thaliana* mesophyll protoplasts and polyethylene glycol- mediated transfection were performed as described in previous [41]. Briefly, well-expanded leaves were cut into 0.5–1 mm leaf strips and dipped into enzyme solution containing 1.5% cellulose ‘Onozuka’ R10 and 0.4% macerozyme R10 (Yakult Pharmaceutical). After 30 min vacuum-infiltration and 3 h digestion at room temperature in the dark, the enzyme-protoplast mixture was filtered with 75-mm nylon mesh. Subsequently, protoplasts were washed twice with W5 buffer and suspended in MMG buffer to a density of 2 × 10^5^ cells/ml. 10 μg plasmid and 100 μL protoplast suspension were used per reaction of transfection. Then the transfection process was stopped with W5 buffer. Finally, protoplasts were incubated in W1 buffer at room temperature in the dark for 12–16 h for gene expression.

### Particle bombardment assays

The plasmid of PvRxLR16 fused with GFP and the GFP vector were mixed with gold power (Bio-Rad, USA), respectively. And then they were transformed into onion epidermal cells using a He⁄ 1000 particle delivery system (Bio-Rad, USA) at 1100 psi as described by Mare et al. [42]. The onion epidermal cells were cultured in Murashige-Skoog (MS) media in darkness at 21 °C for 18 h before observation.

### Confocal microscopy

Patches were cut from onion epidermal cells and agroinfiltrated *N. benthamiana* leaves. Then microscope slides carrying the patches and Arabidopsis protoplasts were imaged using a Nikon C1 Si/TE2000E confocal laser scanning microscope. The excitation and emission wavelengths for GFP were 488 nm and 505–530 nm, respectively.

### VIGS Assays in *N. benthamiana*

*Agrobacterium* strains harboring pTRV1 and pTRV2-gene were grown overnight in LB containing appropriate antibiotics, harvested, suspended in infiltration medium [10 mM MgCl_2_, 10 mM MES (pH 5.7) and 200 μM acetosyringone], mixed in a 1:1 ratio to an OD_600_ = 0.5 for each strain. The cocultures were then infiltrated using needless syringes on the abaxial side of 3-week-old leaves of *N. benthamiana*. The agroinfiltrated plants were then grown for about 3 weeks before cell death assay. Gene silencing levels were checked by quantitative reverse transcription (RT)-PCR.

### RNA extraction and real time quantitative RT-PCR

Total RNA of *N. benthamiana* leaves was isolated using a commercial kit (RNA simple Total RNA Kit, Tiangen) following the recommended protocols. All cDNA synthesis and quantitative RT-PCR reactions were performed according to previous methods [43]. The *EF1α* gene from *N. benthamiana* was used as an internal reference gene to determine relative expression values. Primers were designed by primer 5.0 software (PREMIER Biosoft International, Canada) with the default settings.

### Protein extraction and Western blot

Agroinfiltrated *N. benthamiana* leaves were harvested at 2 dpi and homogenized in liquid nitrogen. And then protein extraction buffer (1 mL) containing 50 mM 4-(2-hydroxyethyl)-1-piperazineëthanesulfonic acid (HEPES), 150 mM KCl, 1 mM ethylenediaminetetraacetate (EDTA) (pH 8.0), 0.1% triton X-100, 1 mM dl-dithiothreitol (DTT), and 1 × Protease Inhibitor Cocktail (Sigma) was added to 500 mg of each ground sample. The samples were mixed and centrifuged at 12000 rpm for 15 min at 4 °C. Supernatants were separated by 12% sodium dodecyl sulfate-polyacrylamide gel electrophoresis (SDS-PAGE) and transferred to a nitrocellulose blotting membrane. Western blot analysis was conducted using an anti-cFlag peroxidase conjugate (Sigma- Aldrich).

### Trypan blue staining

Cell death in *N. benthamiana* leaves was examined by using trypan blue staining. Agroinfiltrated leaves were harvested at 2.5 dpi and soaked in boiling trypan blue solution [10 mL lactic acid, 10 mL glycerol, 10 mL ddH_2_O, 10 g phenol and 20 mg trypan blue (Sigma-Aldrich)] for 5 min and incubated for 3 h. Samples were then decolorized in 2.5 g/mL chloral hydrate solution to clear the background and photographed.

### Oxygen burst detection

Oxygen burst was detected according to H_2_O_2_ accumulation after staining *N. benthamiana* leaves with 3, 3′- diaminobenzidine (DAB) [44]. Briefly, Agroinfiltrated *N. benthamiana* leaves were detached at 72 hpi. Then the leaves were soaked in DAB solution (1 mg/mL ddH_2_O) and maintained for 8 h at 25 °C. The leaf tissues were cleared by boiling in 95% ethanol for 15 min until all the chlorophyll was entirely bleached. The blenched samples were then soaked in trichloracetic aldehyde solution at 2.5 g/mL to further remove the background. The H_2_O_2_ levels were quantified by Image J (National Institutes of Health, USA) and Photoshop (Adobe Systems Software Ireland Ltd). All experiments were repeated three times.

### Electrolyte leakage assays

Cell death of *N. benthamiana* was assayed by determining ion leakage from leaf discs as previously described [27]. For each sample, five leaf discs (8 mm diameter) were placed into a 10-ml tube containing 5 ml sterile and double-distilled water for 3 h at room temperature (RT). Then the conductivity EC1 of the bathing solution was measured with a conductivity meter (DDS-307, Rex Shanghai, China). The conductivity EC2 of the solution was measured by boiling the sealed tubes for 25 min and then cooling to RT. Electrolyte leakage (%) = 100 × EC1/EC2. These experiments were repeated three times.

### Accession numbers

All sequence information from this study can be found in GenBank data library of National Center for Biotechnology Information (NCBI) under accession numbers listed in Additional file 3: Table S2.

## Results

### RxLR16 from *P. viticola* induces cell death in *N. benthamiana*

**Fig. 1:**
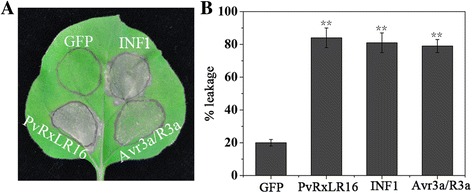
PvRxLR16 induces cell death in *N. benthamiana.* **a** Leaves of *N. benthamiana* were infiltrated with *A. tumefaciens* carrying PVX-*Flag::PvRxLR16* and the indicated controls. The photography was taken 5 d post infiltration. The experiment was repeated three times with similar results. **b** Quantification of cell death by measuring electrolyte leakage. *Error bars* represent standard errors from three biological replicates (^**^, *P* < 0.01, Dunnett’s test)

Our previous analysis identified 51 candidates RxLR effectors from *P. viticola* and only several putative effectors were tested for suppression of BAX- and INF1-induced cell death [38]. To identify defense response elicitor proteins, we expressed all these RxLR proteins transiently by agroinfiltration of PVX vectors and assayed them for induction of cell death in *N. benthamiana* leaves. INF1 from *P. infestans* and AVR3a/R3a, which are known to induce cell death in *N. benthamiana*, were served as positive controls [18, 45]. Of the 51 RxLR effectors, only PvRxLR16 developed a visible phenotype, inducing an HR in *N. benthamiana* 5d after infiltration [39], showing the similar symptom to that induced by INF1 and AVR3a/R3a (Fig. 1a and b). As a negative control, no HR phenotypes were observed in the GFP-infiltrated leaves (Fig. 1a and b). We also expressed PvRxLR16 in *Arabidopsis* and tomato. However, expression of the *PvRxLR16* did not trigger cell death in these two plant species (data not shown). These results demonstrated that PvRxLR16 may be recognized by an endogenous disease resistance protein, resulting in avirulence on *N. benthamiana*.

### PvRxLR16 requires nuclear localization to trigger cell death

**Fig. 2:**
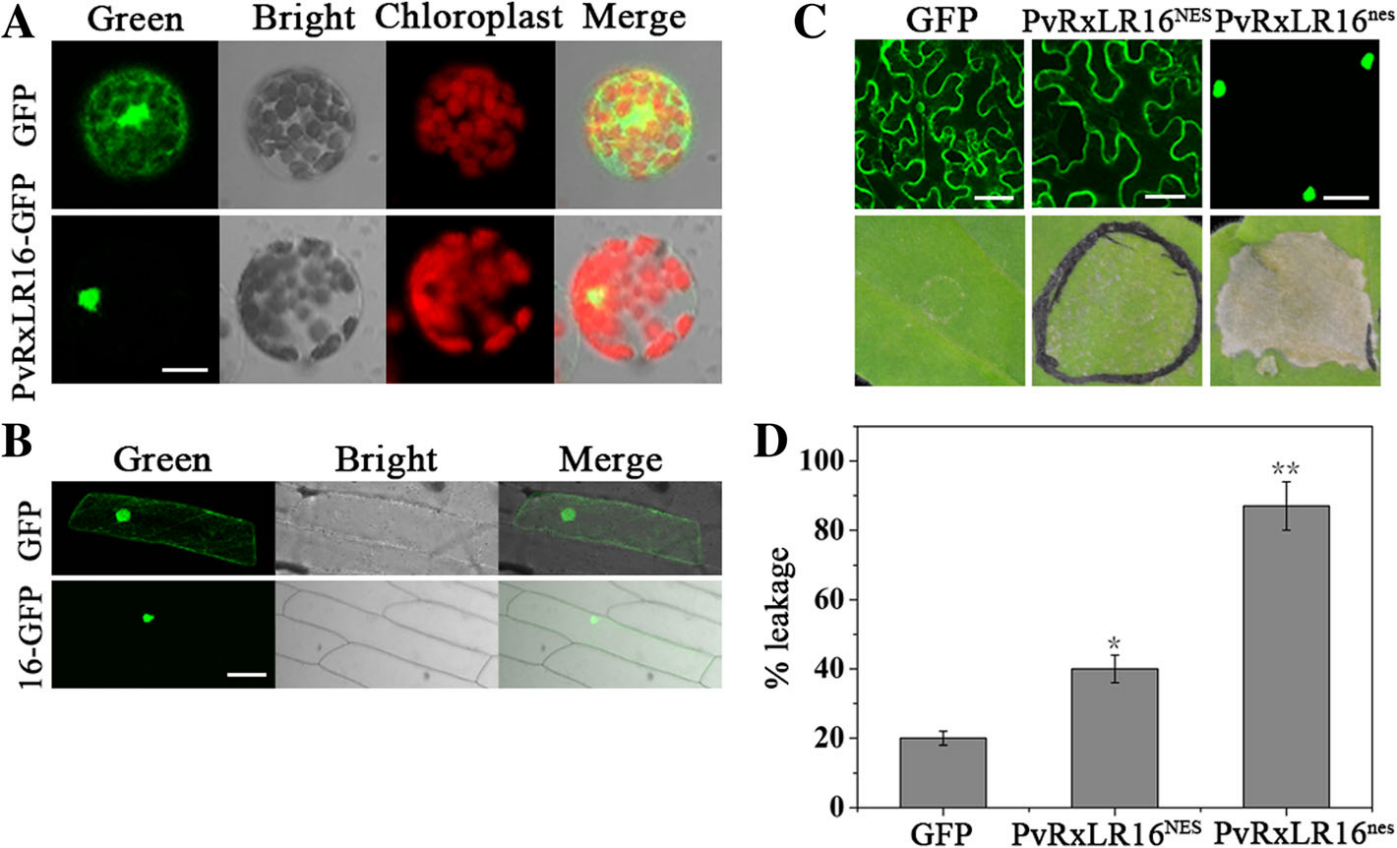
PvRxLR16 effector domains function in plant nucleus. **a** Green fluorescence-tagged PvRxLR16 were transiently expressed in *Arabidopsis* protoplasts via PEG-mediated transformation approach. Scale bar =25 μm. **b** GFP- PvRxLR16 fusion protein was expressed in onion epidermal cells. Scale bar =50 μm. **c** Nuclear localization is required for PvRxLR16-triggering cell death. *N. benthamiana* leaves were agroinfiltrated with the indicated constructs 2 d and 5 d before assessment of GFP confocal imaging and cell death observation, respectively. NES and nes represent the nuclear export signal and nonfunctional NES. Scale bar =20 μm. **d** Quantification of cell death by measuring electrolyte leakage. *Error bars* represent standard errors from three biological replicates (^**^ for *P* < 0.01 and ^*^ for *P* < 0.05, Dunnett’s test)

To determine whether PvRxLR16 localize to nucleus in different plant species as in *N. benthamiana* [39], it was fused with the N terminus of GFP under the control of the CaMV 35S promoter and transiently expressed in *Arabidopsis* protoplasts and onion epidermal cells. Result demonstrated that the green fluorescence almost completely accumulated in the nucleus of *Arabidopsis* protoplasts, while control GFP was detected in both cytoplasm and nucleus (Fig. 2a). When we transiently expressed the PvRxLR16-GFP fusion protein with the pH7FWG2,0 vector in onion epidermal cells, the PvRxLR16 protein was also located in nucleus (Fig. 2b). The combined results from these two experiments clearly concluded that PvRxLR16 localizes to nucleus.

To investigate whether the nuclear localization is required for PvRxLR16 to elicit cell death, a synthetic NES (nuclear exclusion signal) and a nes (nonfunctional NES) [46] were added to the C terminus of PvRxLR16, respectively. The fused proteins were ectopically expressed using agroinfiltration in *N. benthamiana*. PvRxLR16^NES^, excluded from the nucleus, consistently failed to induce cell death at 5 dpi, while PvRxLR16^nes^ robustly induced cell death as the wild-type which retained in the nucleus (Fig. 2c and d). Thus, we inferred that nuclear localization is required for the triggering of cell death by PvRxLR16.

### Functional motifs of PvRxLR16

**Fig. 3:**
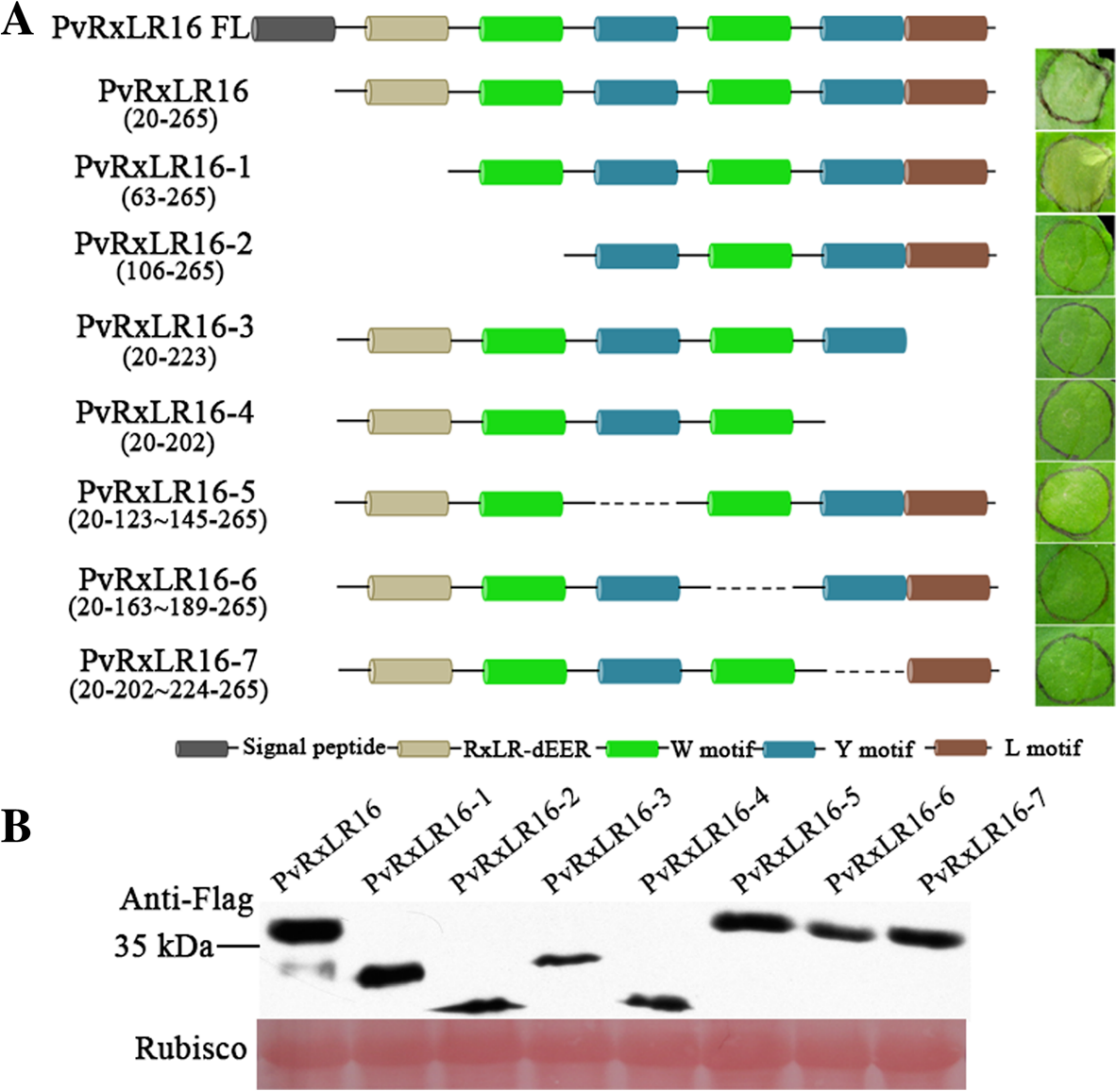
Deletion analysis of PvRxLR16. **a** Left column, schematic diagrams of deletion mutants for PvRxLR16. Right column, deletion mutants of PvRxLR16 were expressed by agroinfiltration in *N. benthamiana* to investigate induction of cell death. The representative pictures were taken at 5 dpi. **b** Immunoblot analysis of proteins from *N. benthamiana* leaves transiently expressing PvRxLR16 and its deletion mutants from a PVX-3 × Flag vector

Sequence analysis showed that full-length PvRxLR16 encodes a polypeptide of 265 amino acids with a predicted signal peptide (SP) (aa 1–18) and a RxLR-dEER motif (aa 46–61). In addition, PvRxLR16 was predicted to contain W/Y/L motifs using the HMMER v3.0 package [47]. To identify the potential functional motifs of PvRxLR16 for induction of cell death, deletion mutants of PvRxLR16 were analyzed using agroinfiltration in *N. benthamiana*. None of the deletion mutants but PvRxLR16–1, a mutant of RxLR-dEER motif deleted, could induce cell death (Fig. 3a). Immunoblot analysis showed that all deletion mutants of PvRxLR16 accumulated to comparable degrees in *N. benthamiana* leaves (Fig. 3b). These data demonstrated that all the W/Y/L motifs are required for inducing cell death.

Identification of putative *N*-glycosylation sites required for the cell death-inducing activity of PvRxLR16.

**Fig. 4:**
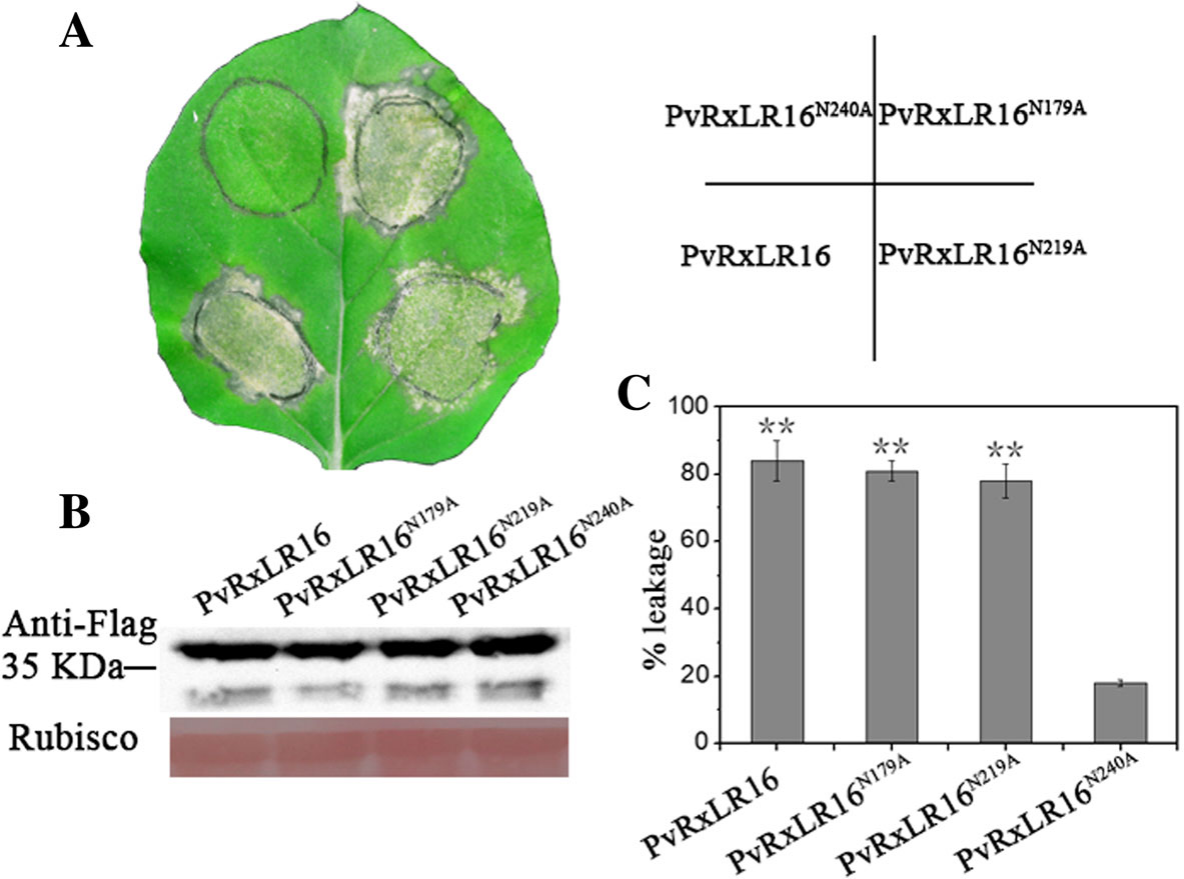
Functional characterization of three putative *N*-glycosylation sites for PvRxLR16. **a** Expression of PvRxLR16 and its mutants in *N. benthamiana* by agroinfiltration. Typical symptoms were photographed at 5 dpi. **b** Immunoblot analysis of proteins from N. benthamiana leaves transiently expressing PvRxLR16 and its site-specific mutations from a PVX-3 × Flag vector. **c** Quantification of cell death by measuring electrolyte leakage. *Error bars* represent standard errors from three biological replicates (^**^, *P* < 0.01, Dunnett’s test)

Secreted proteins often undergo *N*-linked glycosylation which is one of the most ubiquitous posttranslational modifications. It has been reported that *N*-glycosylation is crucial for both the structure and function of effector proteins that play important roles in the pathogenesis of filamentous fungi [48]. Using NetNGlyc1.0 (available online at http://www.cbs.dtu.dk/services/NetNGlyc/), PvRxLR16 is predicted to be a glycoprotein with three putative *N*-glycosylation sites that are located at Asn-170 (NGSS), Asn-219 (NLTT) and Asn-240 (NEST). To establish their contribution to cell death-inducing activity, site-specific mutations were introduced into the three putative *N*-glycosylation sites in the PVX-PvRxLR16-3xFLAG construct. Then they were transiently expressed in leaves of *N*. *benthamiana*. It is obvious that both PvRxLR16^N170A^ and PvRxLR16^N219A^ elicited visible cell death response as wild type PvRxLR16 five days after infiltration (Fig. 4a). Strikingly, PvRxLR16^N240A^ could hardly induce any cell death symptoms (Fig. 4a). All mutations of PvRxLR16 were successfully expressed in *N. benthamiana* (Fig. 4b). Cell death was quantified by measurement of ion leakage [49] (Fig. 4c). This finding highlights the importance of this predicted *N*- glycosylation site Asn-240 for the cell death-inducing activity of PvRxLR16.

### PvRxLR16-triggered cell death in *N. benthamiana* depends on SGT1, Hsp90 and RAR1 but not Serk3/Bak1

**Fig. 5:**
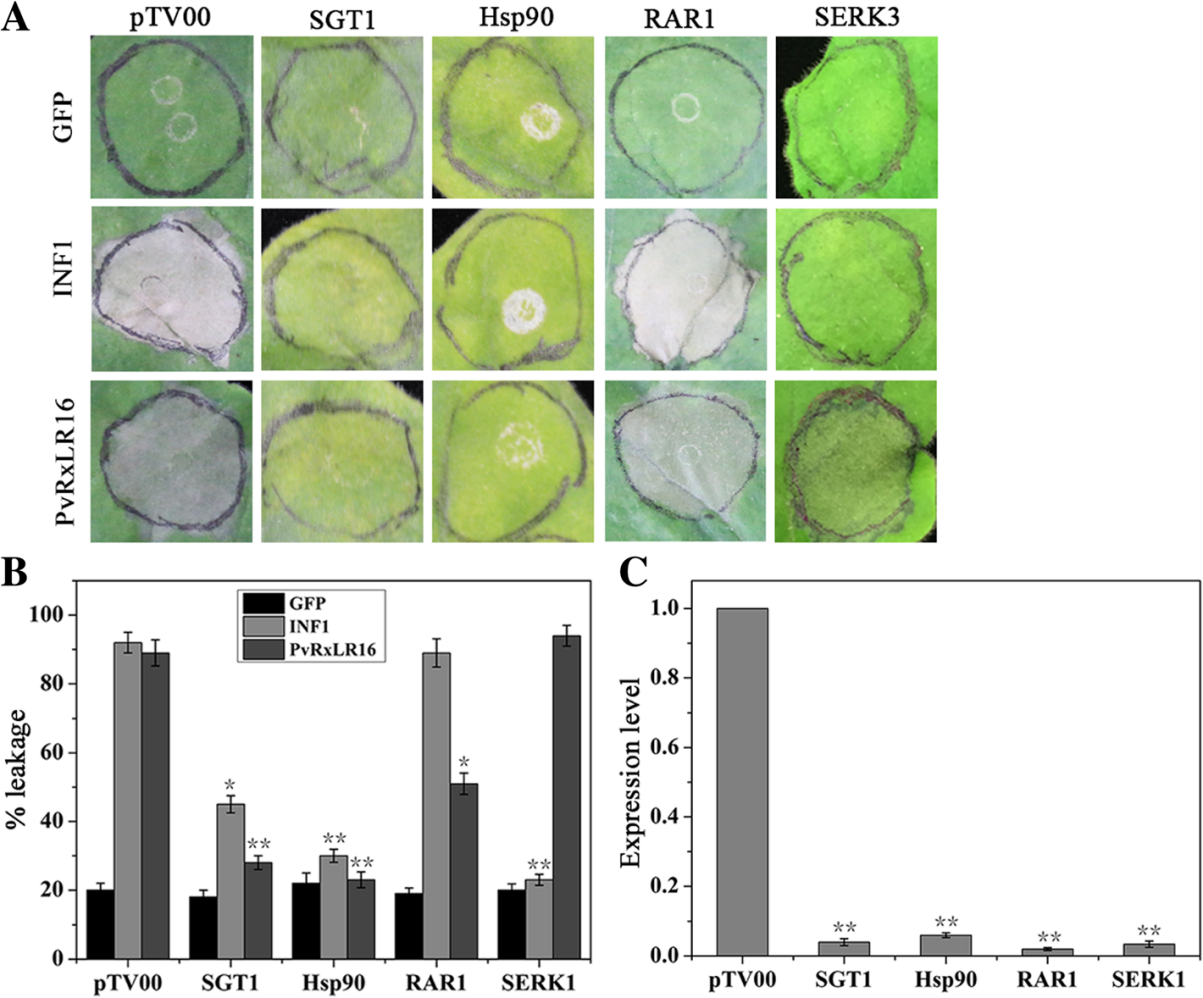
SGT1, Hsp90, and RAR1 were required for PvRxLR16-induced cell death in *N. benthamiana*. **a** PvRxLR16 was transiently expressed in *N. benthamiana* leaves silenced for pTV00 (control), *SGT1*, *Hsp90*, *RAR1*, and *SERK3*. GFP and INF1 are control proteins. Typical symptoms were photographed after 5 d after agroinfiltration. **b** Quantification of cell death by measuring electrolyte leakage. Averages and standard errors were calculated from three independent experimental treatments (^**^ for *P* < 0.01 and ^*^ for *P* < 0.05, Dunnett’s test). **c** Transcript levels of indicated genes in silenced *N. benthamiana* measured by quantitative RT-PCR. *Error bars* represent standard errors from three biological replicates (^**^, *P* < 0.01, Dunnett’s test)

It is known that SGT1, Hsp90 and RAR1 perform essential functions in R protein- mediated HR by regulating the stability of R protein complex [50, 51]. The receptor-like kinase SERK3/BAK1 was identified as crucial factor in various PTI responses, including programmed cell death induction by INF1 [52]. To determine the involvement of SGT1, Hsp90, RAR1 and Serk3 in induction of cell death by PvRxLR16, we performed a VIGS assay against these genes in *N. benthamiana*. The silenced plants were then agroinfiltrated with PvRxLR16 or INF1. Results showed that PvRxLR16 failed to trigger cell death in *SGT1*-, *Hsp90*- and *RAR1*- silenced plants, whereas it was still capable of inducing cell death in *Serk3*-silenced plants (Fig. 5a and b). Reverse transcription- quantitative PCR (RT-qPCR) analysis confirmed that expressions of purpose genes were markedly reduced in silenced plants compared with pTV00, validating successful silencing (Fig. 5c). These results indicate that PvRxLR16-triggered cell death is dependent on SGT1, Hsp90 and RAR1, but independent of Serk3/Bak1.

### Involvement of mitogen-activated protein kinase (MAPK) cascades

**Fig. 6:**
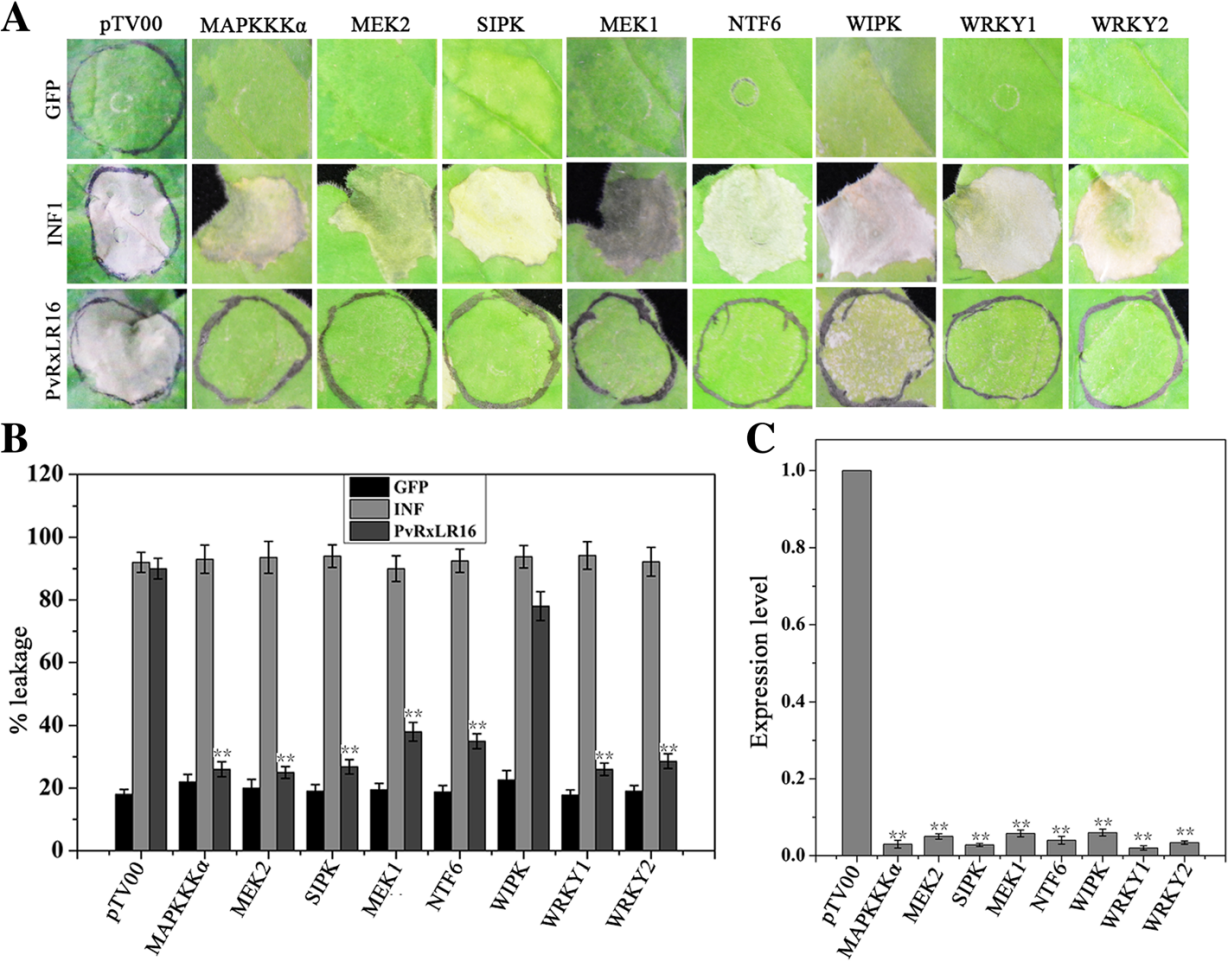
MAPK cascades were required for PvRxLR16-induced cell death in *N. benthamiana*. **a** PvRxLR16 was transiently expressed in *N. benthamiana* leaves silenced for indicated MAPK cascades genes. GFP and INF1 are control proteins. Typical symptoms were photographed after 5 d after agroinfiltration. **b** Quantification of cell death by measuring electrolyte leakage. Averages and standard errors were calculated from three independent experimental treatments (^**^, *P* < 0.01, Dunnett’s test). **c** Transcript levels of indicated genes in silenced *N. benthamiana* measured by quantitative RT-PCR. *Error bars* represent standard errors from three biological replicates (^**^, *P* < 0.01, Dunnett’s test)

MAPK cascades play a remarkably important role in both PTI and ETI [53]. A series of kinases and transcription factors have been characterized that play an essential role in plant immunity and cell death induction during interactions between plants and pathogens, including mitogen-activated protein kinase kinase kinase (MAPKKKα), MAP kinase kinase 2 (MEK2), salicylic acid-induced protein kinase (SIPK), MAP kinase kinase 1 (MEK1), NTF6, wound-induced protein kinase (WIPK), WRYK1 and WRKY2 [54–56]. To investigate the possible roles of these proteins in PvRxLR16-induced cell death, each of them was silenced in *N. benthamiana* via VIGS and challenged with PvRxLR16 effector. INF1 was chosen as a positive control which triggers cell death independently of these kinases and transcription factors. Cell death was compromised in plants silenced for all genes with one exception (WIPK) (Fig. 6a and b). Figure 6c showed the transcript abundances of purpose genes in the silenced *N. benthamiana*, validating successful silencing. These results suggest that most mitogen-activated protein kinases and transcription factors tested in this study are involved in the perception of PvRxLR16 by *N. benthamiana*.

### PvRxLR16 induces disease resistance in *N. benthamiana* and promotes ROS accumulation

**Fig. 7:**
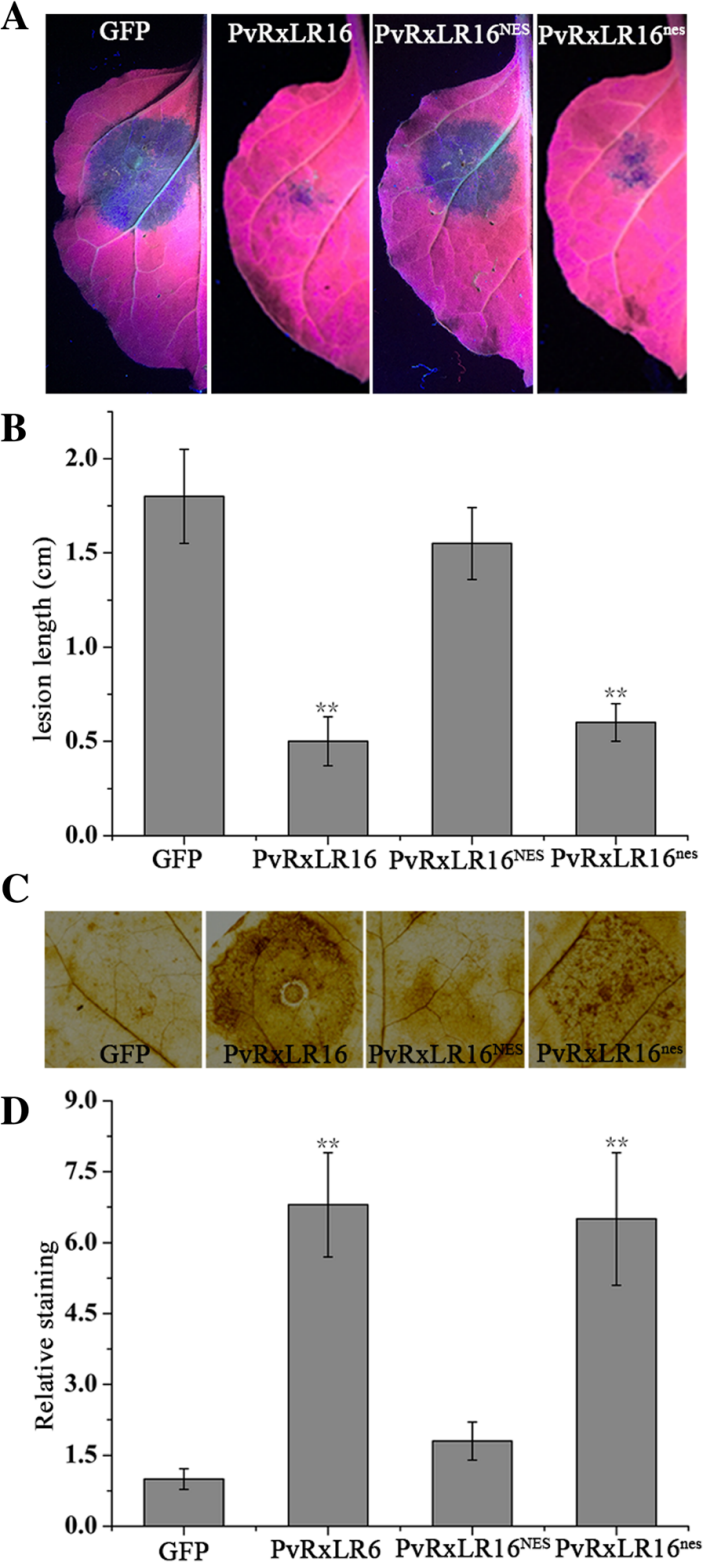
Introduction of disease resistance and H_2_O_2_ accumulation by PvRxLR16 in *N. benthamiana*. **a** Lesions of the *N. benthamiana* leaves expressing the indicated genes inoculated with *P. capsici* at 36 hpi. **b** Lesion diameters of *N. benthamiana* leaves (**P < 0.01, Dunnett’s test). **c** DAB staining of the *N. benthamiana* leaves at 3 dpi expressing the indicated genes. **d** The relative levels of DAB staining. *Asterisks* indicate significant differences (**P < 0.01, Dunnett’s test). These experiments were replicated three times with six leaves per biological replicate

To determine whether PvRxLR16 could activate other defense responses in *N. benthamiana*, plants were agroinfiltrated separately with PvRxLR16, PvRxLR16^NES^, or PvRxLR16^nes^, and GFP was used as a control. The final concentration of *Agrobacterium* suspension was adjusted to an OD_600_ of 0.2 which could not induce cell death at 2.5 d post-infiltration (Additional file 4: Figure S2). And then 24 h later, the infiltrated regions were inoculated with *P. capsici* zoospores and disease development was evaluated by measuring lesion diameter of disease at 36 hpi. Disease symptoms became visible on leaves agroinfiltrated with GFP expression constructs, which was similar to PvRxLR16^NES^, whereas PvRxLR16 and PvRxLR16^nes^-infiltrated leaves were protected against pathogen infection (Fig. 7a). Determination of *P. capsici* lesion diameter (Fig. 7b) revealed that PvRxLR16 and PvRxLR16^nes^ decreased *P. capsici* growth significantly.

To explore mechanisms behind the increased disease resistance of PvRxLR16, the production of H_2_O_2_ in infected leaves was analysed using the diaminobenzidine (DAB) staining. The relative staining was significantly higher in infected regions of PvRxLR16 and PvRxLR16^nes^ compared to that in the PvRxLR16^NES^ and GFP control (Fig. 7c and d), indicating that PvRxLR16 needs to target the plant nucleus to promote H_2_O_2_ accumulation. Taken together, these data showed that PvRxLR16 requires nuclear localization to activate plant defenses and prevent colonization of the pathogen.

### PvRxLR16 enhances the expression of defence-associated genes in *N. benthamiana*

**Fig. 8:**
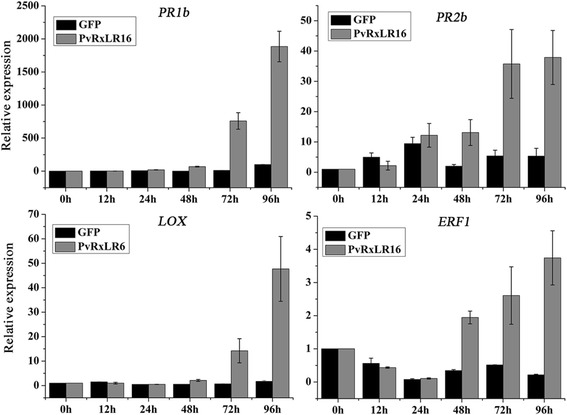
Upregulation of defense- related genes mediated by PvRxLR16 in *N. benthamiana.* Transcript level of the *PR1b*, *PR2b*, *LOX* and *ERF1* genes induced by PvRxLR16 at different time points. Means and standard errors from three independent replicates are shown

To increase our understanding of the role of PvRxLR16 in plant defense responses, transcriptional levels of defence-related genes were analysed using real time qRT-PCR. Since salicylic acid (SA)-, jasmonate acid (JA)-, and ethylene (ET)-mediated signal transduction pathways play critical roles in disease resistance to pathogens [57], expression levels of marker genes for each pathway were analysed. The *PR1a/PR2b*, *ERF1* and *LOX* are marker genes for salicylate-, ethylene- and jasmonate -mediated signaling pathway, respectively [58–60]. Expression levels of these defence-related genes were significantly upregulated in the leaves transiently expressing PvRxLR16 compared to GFP (Fig. 8). These results suggest that expression of PvRxLR16 may enhance the expressional levels of defence-associated genes in *N. benthamiana,* resulting in disease resistance.

### PvRxLR16-triggered immunity can be suppressed by other PvRxLR effectors

**Fig. 9:**
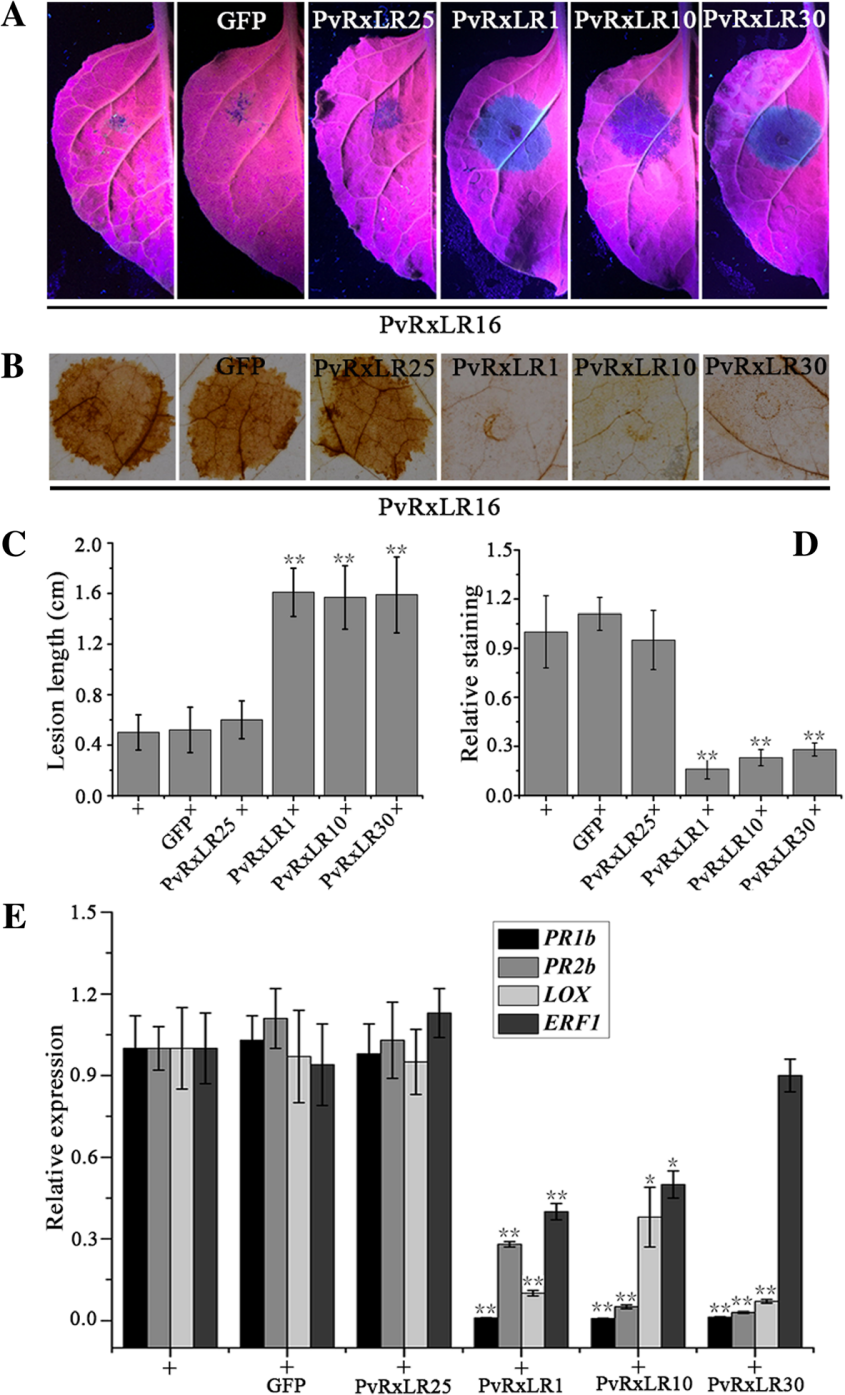
Suppression of PvRxLR16-induced disease resistance and immune responses by PvRXLR effectors in *N. benthamiana*. **a** Lesions of the *N. benthamiana* leaves expressing the indicated genes inoculated with *P. capsici* at 36 hpi. The leaves were infiltrated with PvRxLR16 constructs 12 h after expressing each PvRxLR or GFP using agroinfiltration. Then the infiltrated leaves were inoculated with *P. capsici* 48 h after expression of PvRxLR16. The representative pictures were taken at 36 hpi post infection of *P. capsici*. **b** DAB staining of the *N. benthamiana* leaves at 3 dpi expressing *PvRxLR16*. PvRxLR effectors (PvRxLR1, 10 and 30) and GFP were transiently expressed in *N. benthamiana* leaves by agroinfiltration 12 h before infiltration of PvRxLR16. **c** Lesion diameters of *N. benthamiana* leaves (**P < 0.01, Dunnett’s test). **d** The relative levels of DAB staining. Significant differences based on Dunnett’s test are indicated by the *asterisks* (**P < 0.01). These experiments were replicated three times with six leaves per biological replicate. **e** Relative expression levels of *PR1b*, *PR2b*, *LOX*, and *ERF1* genes induced by PvRxLR16 were suppressed by other PvRxLRs (PvRxLR1, 10 and 30) in *N. benthamiana*. PvRxLR effectors (PvRxLR1, 10 and 30) and GFP were transiently expressed in *N. benthamiana* leaves by agroinfiltration 12 h before infiltration of PvRxLR16. The relative transcript levels of defense-related genes were detected at 3 dpi expressing *PvRxLR16* (+). *EF1a* was used as an endogenous control. Means and standard errors from three independent replicates are shown. Significant differences based on Dunnett’s test are indicated by the asterisks (**P < 0.01, *P < 0.05)

Although individual effectors can trigger strong defense responses, some other RxLR effectors are capable of inhibiting defense responses including effector- triggered immunity [61]. Previous data have shown that most PvRxLRs tested can suppress cell death induced by PvRxLR16 [39]. To further determine whether PvRxLR16-induced defense responses in addition to cell death could also be repressed by PvRxLR effectors, we assayed whether disease resistance, ROS production and defence-related genes activated by PvRxLR16 could be repressed by transiently expressing *PvRxLR1*, *PvRxLR10*, *PvRxLR30* and *PvRxLR25* in *N. benthamiana*. The leaves were infiltrated with PvRxLR16 constructs 12 h after expressing each PvRxLR or GFP using agroinfiltration. ROS accumulation was detected by staining of leaves 72 h after expressing PvRxLR16. For disease development assay, the infiltrated leaves were inoculated with *P. capsici* zoospores (10 μl, 100 zoospores μl^−1^), and lesion diameter of disease was measured at 36 hpi. Results demonstrated that PvRxLR1, PvRxLR10 and PvRxLR30 suppressed PvRxLR-16 induced disease resistance (Fig. 9a and c) and ROS accumulation (Fig. 9b and d) compared with negative control PvRxLR25 and GFP. QRT-PCR analysis revealed that these three PvRxLRs could also reduce the introduction of defence-related genes by PvRxLR16 to some extent (Fig. 9e). Therefore, the defense responses of *N. benthamiana* elicited by PvRxLR16 could be efficiently repressed by other PvRxLR effectors.

## Discussion

In plant-pathogen interactions, RxLR effectors play a vital role in the establishment of pathogen infection and also conversely a role in plant defense [26]. In recent years, hundreds of RxLR effectors were predicated in various oomycete pathogen species via genome sequencing or RNA-seq [25, 38, 62–66]. Large-scale functional surveys of candidate RxLR effectors from *P. infestans*, *P. sojae* and *H. arabidopsidis* revealed that majority of them could suppress cell death and immunity, whereas a few could trigger cell death or immune responses [21, 61, 67]. Some cell death-inducing effectors, such as PsAvh163 and PsAvh241 from *P. sojae*, have been proven to activate ETI or PTI in plant [27, 68]. The candidate effector PvRxLR16 was identified as the only one that can elicit cell death in *N. benthamiana* in a survey of *P. viticola* effectors [39]. Here we also demonstrate that it also induce other immune responses in addition to cell death. The defense responses triggered by PvRxLR16 has the potential to block infection of *N. benthamiana* by *P. capsici*, while in the mean time, other PvRxLR effectors have the capacity to suppress the PvRxLR16-triggered immune responses.

The species specific cell death and disease resistance induction of PvRxLR16 indicates that it seems to function as a typical avirulence protein in *N. benthamiana*. PvRxLR16 could still trigger cell death in *BAK1* (*Serk3*)-silenced *N. benthamiana*, indicating that it acts independently of the detection of cell surface pattern recognition receptors. However, triggering of cell death in *N. benthamiana* requires SGT1, HSP90 and RAR1, suggesting that PvRxLR16-dependent cell death may result from ETI rather than non-specific toxicity of PvRxLR16. Additionally, silencing of MAPK and transcription factor genes indicated that PvRxLR16-induced cell death requires all tested genes with one exception, WIPK, which is required for transducing signals from the PAMP receptor flagellin-sensitive 2 (FLS2) [27]. Collectively, we speculate that PvRxLR16 may targets an upstream component of plant ETI pathway, possibly an endogenous R protein. The endogenous protein responsible for the detection may have evolved to recognize homologous genes of PvRxLR16 in a downy mildew pathogen of *Nicotiana* species (e.g. *Peronospora tabacina* Adam).

A growing body of evidence indicates that plant cell nucleus is a main target for oomycete pathogen effectors. A recent study that investigated the subcellular localization patterns of *H. arabidopsidis* RxLR effector candidates in planta found that 66% RxLR effectors could target the host cell nucleus [28]. Moreover, functional analysis of CRN effectors from *Phytophthora* confirmed that majority of them target the nucleus [40, 69, 70]. The authors showed that alteration of nuclear targeting signals in several CRN effectors prevented their cell death-inducing activities. In this study, we demonstrated that nuclear localization of PvRxLR16 is required to induce cell death and defence responses. A similar case was reported in *P. infestans* where the avirulence factor AVR1 has also to be present in nucleus to activate *R* gene-mediated resistance [35]. Therefore, it is reasonable to believe that the nucleus localization of PvRxLR16 is essential for endogenous proteins recognition, resulting in immune responses.

Effector-triggered immunity (ETI) is generally associated with pathogen resistance, induction of ROS burst, ion influx, and increased expression levels of defence-related genes [71]. In this study, we observed that transient expression of the *PvRxLR16* in *N. benthamiana* enhanced resistance against *P. capsici*, and also ROS accumulation and expression level of defence-related genes were increased. These defence responses were consistent with ETI. It is worth noting that SA, JA and ET signaling pathways were all activated by PvRxLR16 effector. It is plausible that PvRxLR16 could elicit multiple defense pathways to restrict pathogen growth. SA, JA and ET are the classical immunity hormones which play key roles in plant defenses against pathogens. SA signaling is generally crucial for immunity against biotrophs or hemibiotrophs, while JA and ET signaling are often important for immunity against necrotrophs, although there are exceptions [57]. It will be very interesting to determine whether PvRxLR16-induced immunity can inhibit invasion of different pathogens to *N. benthamiana*.

Although plants have the ability to detect and respond to RxLR effectors, successful pathogens have clearly evolved sophisticated mechanisms to surmount the immune responses of hosts [72]. Interference with PTI and ETI by effectors secreted by pathogens has been reported in multiple systems [34]. A complex repertoire of candidate RxLR effectors was identified from *P. viticola*, and expression pattern of which could be grouped into four main groups. Eighteen PvRxLR effectors from each of the different expression profile groups could suppress cell death induced by PvRxLR16 in *N. benthamiana* with several exceptions [39]. All of these RxLR effectors could also inhibit cell death induced by BAX, INF1, PsojNIP, PsCRN63 and Avr3a/R3a, indicating that they all may target a common pathway that mediates plant cell death and defense responses. Several of these effectors, particularly *PvRxLR1*, *PvRxLR10*, and *PvRxLR30*, were expressed mediumly or highly during early infection and showed similar expression pattern as PvRxLR16 [39], suggesting that these RxLR effectors may act preemptively to inhibit the capability of hosts to respond to PvRxLR16. However, these effectors could not suppress PvRxLR16-triggered immune responses when PvRxLR16 is already highly expressed in plant, prior to induction of these three RxLR effectors (data not shown). Thus, the capability of plants to respond to the PvRxLR16 will probably place an upper limit on the expression level of the effector by *P. viticola*. Isolation of *PvRxLR16* from several physiological races of *P. viticola* revealed no sequence polymorphism in *PvRxLR16* (data not shown). Unexpectedly, this effector does not appear to be under diversifying selection though it can induce cell death and defence responses in *N. benthamiana*. It is currently unknown whether PvRxLR16 can activate cell death under natural conditions in its host, grapevine. PvRxLR16 may possibly not trigger cell death during physiological infection, alleviating selection pressure for changes in itself. Unfortunately, since *P. viticola* can hardly be genetically modified because of its typical obligate lifestyle, it is difficult to determine whether PvRxLR16 is essential for the pathogenicity of *P. viticola* during infection.

## Conclusions

This is the first study to report that a putative RxLR effector from *P. viticola* is recognized by the plant immune system and even may act as an avirulence gene in *N. benthamiana*. The ability of PvRxLR16 to trigger cell death will be very useful to exploit ATTA (*Agrobacterium tumefaciens*-mediated transient expression), VIGS or other tools in *N.benthamiana*. Moreover, it may be also used for identifying novel defence related genes against *P. viticola* or other oomycetes which will be pivotal for providing insights into novel disease control strategies.

## Additional files

Additional file 1: Table S1. Primers used in this study. (DOCX 24 kb)

Additional file 2: Figure S1. Schematic diagrams of constructs used in this study. (TIFF 1018 kb)

Additional file 3: Table S2. Genbank accession numbers of genes in *N. benthamiana* and PvRxLR effectors used in this study. (DOCX 14 kb)

Additional file 4: Figure S2. Transient expression of PvRxLR16 and GFP in *N. benthamiana.* Representative *N. benthamiana* leaves infiltrated with various concentrations (OD_600_ = 0.4, 0.2, 0.1) of *Agrobacterium* suspension containing the PVX-PvRxLR16 or PVX-GFP. Upper pictures, directly photographed 2.5 d post-infiltration. Lower pictures, photographed 2.5 d post-infiltration after staining with trypan blue. (TIFF 1612 kb)

## Abbreviations

ATTA: *Agrobacterium tumefaciens*-mediated transient expression
Avr: Avirulence
DAB: 3, 3′- diaminobenzidine
DTT: Dl-dithiothreitol
EDTA: ethylenediaminetetraacetate
ET: Ethylene
ETI: Effector-triggered immunity
ETS: Effector-triggered susceptibility
FLS2: Flagellin-sensitive 2
GFP: Green fluorescent protein
HEPES: 4-(2-hydroxyethyl)-1-piperazineëthanesulfonic acid
HR: Hypersensitive response
HSP90: Heat shock protein 90
JA: Jasmonate acid
LB: Luria-Bertani
MAPK: Mitogen-activated protein kinase
MAPKKKα: Mitogen-activated protein kinase kinase kinase
MEK1: MAP kinase kinase 1
MEK2: MAP kinase kinase 2
MS: Murashige-Skoog
NCBI: National Center for Biotechnology Information
nes: nonfunctional nuclear exclusion signal
NES: Nuclear exclusion signal
PAMPs/ MAMPs: Pathogen- or microbe-associated molecular patterns
PCD: Programmed cell death
PRRs: Pattern recognition receptors
PTI: PAMP-triggered immunity
RAR1: Required for Mla12 resistance
ROS: Reactive oxygen species
SA: Salicylic acid
SDS-PAGE: Sodium dodecyl sulfate-polyacrylamide gel electrophoresis
SERK3: Somatic embryogenesis receptor-like kinase
SGT1: Suppressor of G-two allele of Skp1
SIPK: Salicylic acid-induced protein kinase
SP: Signal peptide
TAIR: The Arabidopsis Information Resource
VIGS: Virus induced gene silencing
WIPK: Wound-induced protein kinase
