# Supplementary material for: A candidate RxLR effector from Plasmopara viticola can elicit immune responses in Nicotiana benthamiana
Source: BMC Plant Biol. 2017 Apr 14;17:75. doi: 10.1186/s12870-017-1016-4 (PMC5391559; doi:10.1186/s12870-017-1016-4)
Supplement: Supplementary file 1 — Primers used in this study. (DOCX 24 kb) [file 12870_2017_1016_MOESM1_ESM.docx]

**Additional file 1: Table S1. Primers used in this study**

| **Primer name** | **Primer sequence** | **Purpose or vector** |
| --- | --- | --- |
| ***NbMAPKKKα-XmaIF***  ***NbMAPKKKα-KpnIR*** | TCCCcccgggAGACCTTCAGTGGGAAATGATC | pTRV2 |
|  | GGggtaccTTGCCTTTCTTCCATTTTGACATAT |  |
| ***NbNTF6-XmaIF***  ***NbNTF6-KpnIR*** | TCCCcccgggGATATAGTTAGACCACCAGACAGA | pTRV2 |
|  | GGggtaccTCTTTACGAGTTCCATCAAAATGC |  |
| ***NbWRKY1-XmaIF***  ***NbWRKY1-KpnIR*** | TCCCcccgggGGATGATACTCCACCTACTAAGTC | pTRV2 |
|  | GGggtaccACATGTTTGTTTTCACCTGATTCC |  |
| ***NbWRKY2-XmaIF***  ***NbWRKY2-KpnIR*** | TCCCcccgggGAAGCAGCTTCTTCTGCTTCTC | pTRV2 |
|  | GGggtaccCGTAGCAGTGCTATAGGTTGCTGT |  |
| ***NbWIPK-XmaIF***  ***NbWIPK-KpnIR*** | TCCCcccgggCATGAAAATGTAATTGGTTTAAGA | pTRV2 |
|  | GGggtaccAACCGACAGACCAAACATCTATAG |  |
| ***NbSIPK-XmaIF***  ***NbSIPK-KpnIR*** | TCCCcccgggGTGATTTAAAGATATGCGATTTTG | pTRV2 |
|  | GGggtaccCTGGGATCAAATGTCAGCATTGTC |  |
| ***NbMEK1-XmaIF***  ***NbMEK1-KpnIR*** | TCCCcccgggGGAGCTATTTCTCTGGTTCTTGAA | pTRV2 |
|  | GGggtaccCCTCCGACTGTATGTATGGAAAAC |  |
| ***NbMEK2-XmaIF***  ***NbMEK2-KpnIR*** | TCCCcccgggTTCTTCCTCGTCGTCTTC | pTRV2 |
|  | GGggtaccCGCCTGTGGAGATAATAGA |  |
| ***Nb* *SGT1-XmaIF***  ***Nb* *SGT1- KpnIR*** | TCCCcccgggGAGCTTTTCGCCGACCGTGCTCAGG | pTRV2 |
|  | GGggtaccGGCACATCAATGCTAACACTAAGTA |  |
| ***NbHsp90-XmaIF***  ***NbHsp90-KpnIR*** | TCCCcccgggGGTGATTAAGGATGTTCTAGGTGAC | pTRV2 |
|  | GGggtaccGTCAACTTCCTCCATCTTGCTGCCC |  |
| ***NbRAR1-XmaI***  ***NbRAR1-KpnI*** | TCCCcccgggGTGGAGTTGTTGCAAGAAAAG | pTRV2 |
|  | GGggtaccATGGAAGATAGCGGGGCCAG |  |
| ***NbSERK3-XmaIF***  ***NbSERK3- KpnIR*** | TCCCcccgggTTGTGCTTCTACAGCCATTCCTGC | pTRV2 |
|  | GGggtaccGGGTCAAAGGACTTCTGAAGGACA |  |
| ***PvRxLR16-XmaIF***  ***PvRxLR16-SalIR*** | TCCCcccgggATGACAGGAAATGCCAAATCG | PGR107 |
|  | ACGCgtcgacCCTATTGAATAAAGGGAA |  |
| ***PvRxLR16-1-XmaIF***  ***PvRxLR16-1-SalIR*** | TCCCcccgggATGGATGCAACTGTT | PGR107 |
|  | ACGCgtcgacCCTATTGAATAAAGGGAA |  |
| ***PvRxLR16-2-XmaIF***  ***PvRxLR16-2-SalIR***  ***PvRxLR16-3-XmaIF***  ***PvRxLR16-3-R***  ***PvRxLR16-3-F*** | TCCCcccgggATGTCGGCAATAGAT | PGR107  PGR107 |
|  | ACGCgtcgacCCTATTGAATAAAGGGAA  TCCCcccgggATGACAGGAAATGCC  ATGGTCTTTGTAGTCTTCGAAGGTCGTGAGATTTTT  TATTTTAAAAATCTCACGACCTTCGAAGACTACAAA |  |
| ***PvRxLR16-3-SalIR***  ***PvRxLR16-4-XmaIF*** | ACGCgtcgacCCTATTGAATAAAGGGAA | PGR107 |
|  | TCCCcccgggATGACAGGAAATGCC |  |
| ***PvRxLR16-4-R***  ***PvRxLR16-4-F*** | ATGGTCTTTGTAGTCTTCGAATGGGCCCAATGCCCT |  |
|  | TTGGAAAGGGCATTGGGCCCATTCGAAGACTACAAA |  |
| ***PvRxLR16-4-SalIR*** | ACGCgtcgacCCTATTGAATAAAGGGAA |  |

| **Primer name** | **Primer sequence** | | | | | **Purpose or vector** |
| --- | --- | --- | --- | --- | --- | --- |
| ***PvRxLR16-5-XmaIF*** | TCCCcccgggATGACAGGAAATGCC | | | | | PGR107 |
| ***PvRxLR16-5-R*** | ATGGTCTTTGTAGTCTTCGAAGTACATCAGTACCGC | | | | |  |
| ***PvRxLR16-5-F*** | ACAGAGGCGGTACTGATGTACTTCGAAGACTACAAA | | | | |  |
| ***PvRxLR16-5-SalIR*** | ACGCgtcgacCCTATTGAATAAAGGGAA | | | | |  |
| ***PvRxLR16-6-XmaIF*** | TCCCcccgggATGACAGGAAATGCC | | | | | PGR107 |
| ***PvRxLR16-6-R*** | CATATGAGGAATGTTGAGAAAAGCGCGGTCTGGAGT | | | | |  |
| ***PvRxLR16-6-F*** | TTGGCGACTCCAGACCGCGCTTTTCTCAACATTCCT | | | | |  |
| ***PvRxLR16-6-SalIR*** | ACGCgtcgacCCTATTGAATAAAGGGAA | | | | |  |
| ***PvRxLR16-7-XmaIF*** | TCCCcccgggATGACAGGAAATGCC | | | | | PGR107 |
| ***PvRxLR16-7-R*** | GTCATCCGCACTCGTCCTCTCGTACATCAGTACCGC | | | | |  |
| ***PvRxLR16-7-F*** | ACAGAGGCGGTACTGATGTACGAGAGGACGAGTGCG | | | | |  |
| ***PvRxLR16-7-SalIR*** | ACGCgtcgacCCTATTGAATAAAGGGAA | | | | |  |
| ***PvRxLR16-NES-XmaIF*** | TCCCcccgggATGACAGGAAATGCC | | | | | PGR107 |
| ***PvRxLR16-NES-SalIR*** | ACGCgtcgacCTACTTGTTAATATCAAGTCCAGCCAA  CTTAAGAGCAAGCTCGTTCCTATTGAATAAAGGGAA | | | | |  |
| ***PvRxLR16-nes-XmaIF*** | TCCCcccgggATGACAGGAAATGCC | | | | | PGR107 |
| ***PvRxLR16-nes-SalIR*** | ACGCgtcgacCTTGTTAGCATCTGCTCCAGCTGCCTTA  AGAGCAAGCTCGTTCCTATTGAATAAAGGGAA | | | | |  |
| ***PvRxLR16-N179A-XmaIF*** | TCCCcccgggATGACAGGAAATGCC | | | | | PGR107 |
| ***PvRxLR16-N179A-R*** | ACTTGACCCCGCTTTCCAGTTAACCAT | | | | |  |
| ***PvRxLR16-N179A-F*** | ATGGTTAACTGGAAAGCGGGGTCAAGT | | | | |  |
| ***PvRxLR16-N179A-SalIR*** | ACGCgtcgacCCTATTGAATAAAGGGAA | | | | |  |
| ***PvRxLR16-N219A-XmaIF*** | TCCCcccgggATGACAGGAAATGCC | | | | | PGR107 |
| ***PvRxLR16-N219A-R*** | GGTCGTGAGCGCTTTAAAATACTCATC | | | | |  |
| ***PvRxLR16-N219A-F*** | GATGAGTATTTTAAAGCGCTCACGACC | | | | |  |
| ***PvRxLR16-N219A-SalIR*** | ACGCgtcgacCCTATTGAATAAAGGGAA | | | | |  |
| ***PvRxLR16-N240A-XmaIF*** | TCCCcccgggATGACAGGAAATGCC | | | | | PGR107 |
| ***PvRxLR16-N240A-R*** | TGTACTTTCCGCCGTACATTCTGCCGG | | | | |  |
| ***PvRxLR16-N240A-F*** | CCGGCAGAATGTACGGCGGAAAGTACA | | | | |  |
| ***PvRxLR16-N240A-SalIR*** | ACGCgtcgacCCTATTGAATAAAGGGAA | | | | |  |
| ***PvRxLR16-attB1-F*** | GGGGACAAGTTTGTACAAAAAAGCAGGCTCTAT  GACAGGAAATGCC | | | | | pH7FWG2 |
| ***PvRxLR16-attB2-R*** | GGGGACCACTTTGTACAAGAAAGCTGGGTCCCT  ATTGAATAAAGGGAA | | | | |  |
| ***PvRxLR16-NES-attB1-F*** | GGGGACAAGTTTGTACAAAAAAGCAGGCTCTAT  GACAGGAAATGCC | | | | | pH7FWG2 |
| ***PvRxLR16-NES-attB2-R*** | GGGGACCACTTTGTACAAGAAAGCTGGGTCCTA  CTTGTTAATATCAAG | | | | |  |
| ***PvRxLR16-nes-attB1-F*** | GGGGACAAGTTTGTACAAAAAAGCAGGCTCTAT  GACAGGAAATGCC | | | | | pH7FWG2 |
| ***PvRxLR16-nes-attB2-R*** | GGGGACCACTTTGTACAAGAAAGCTGGGTCCTTG  TTAGCATCTGCTCC | | | | |  |
| **Primer name** | | | **Primer sequence** | **Purpose or vector** | | |
| ***Nb-EF1α-F*** | | | AGAGGCCCTCAGACAAAC | qRT-PCR | | |
| ***Nb-EF1α-R*** | | | TAGGTCCAAAGGTCACAA |  |  |  |
| ***Nb-PR1b-F*** | | | GTGGACACTATACTCAGGTG | qRT-PCR | | |
| ***Nb-PR1b-R*** | | | TCCAACTTGGAATCAAAGGG |  |  |  |
| ***Nb-PR2b-F*** | | | AGGTGTTTGCTATGGAATGC | qRT-PCR | | |
| ***Nb-PR2b-R*** | | | TCTGTACCCACCATCTTGC |  |  |  |
| ***Nb-ERF1-F*** | | | GCTCTTAACGTCGGATGGTC | qRT-PCR | | |
| ***Nb-ERF1-R*** | | | AGCCAAACCC TAGCTCCATT |  |  |  |
| ***Nb-LOX-F*** | | AAAACCTATGCCTCAAGAAC | | | qRT-PCR | |
| ***Nb-LOX-R*** | | ACTGCTGCATAGGCTTTGG | | |  |  |
| ***NbMAPKKKα-F***  ***NbMAPKKKα-R*** | | ACAACTACCACATCATCAC  ACTACTCACATCATTATCCAAT | | | qRT-PCR  qRT-PCR | |
| ***NbNTF6-F***  ***NbNTF6-R*** | | ATATAGTTAGACCACCAGACA  CCTGTGAAGAGCGTATTATC | | |  |  |
| ***NbWRKY1-F***  ***NbWRKY1-R*** | | TGATACTCCACCTACTAAG  TGACCATCGTTGACTATA | | | qRT-PCR  qRT-PCR | |
| ***NbWRKY2-F***  ***NbWRKY2-R*** | | GCTTCTTCTGCTTCTCAC  CCATCTATAACCATCATCCAA | | |  |  |
| ***NbWIPK-F***  ***NbWIPK-R*** | | ACGAGAATATGACGGAAT  ATAGCAGCAGTGTAATCT | | | qRT-PCR  qRT-PCR | |
| ***NbSIPK-F***  ***NbSIPK-R*** | | CTGCGTCTTATTATGGAGTTG  GAGGAAGTTGTCGGATGT | | |  |  |
| ***NbMEK1-F***  ***NbMEK1-R*** | | TTCTCTGGTTCTTGAATA  TATGTCTCTGTGGATAAC | | | qRT-PCR  qRT-PCR | |
| ***NbMEK2-F***  ***NbMEK2-R*** | | TTAGATGTCACGATATGT  TGTGGAGATAATAGAGTC | | |  |  |
| ***Nb* *SGT1-F***  ***Nb* *SGT1- R*** | | GAGGTGGTGGTGACTATA  CACATCAATGCTAACACTAAG | | | qRT-PCR  qRT-PCR | |
| ***NbHsp90-F***  ***NbHsp90-R*** | | AACGATAAGTCTGTCAAG  CAATCCTCATCAATACTCA | | |  |  |
| ***NbRAR1-F***  ***NbRAR1-R*** | | GATGAAGAAGTGGAGTTG  TACATTGGTCGTAGAAGT | | | qRT-PCR | |
| ***NbSERK3-F***  ***NbSERK3- R*** | | GCGAATGATGATGATGTC  GTTCCACCTCTTCTTCTT | | | qRT-PCR | |
|  | |  | | |  |  |
|  | |  | | |  | |
