# Supplementary figures and images for: A candidate RxLR effector from Plasmopara viticola can elicit immune responses in Nicotiana benthamiana
Source: BMC Plant Biol. 2017 Apr 14;17:75. doi: 10.1186/s12870-017-1016-4 (PMC5391559; doi:10.1186/s12870-017-1016-4)

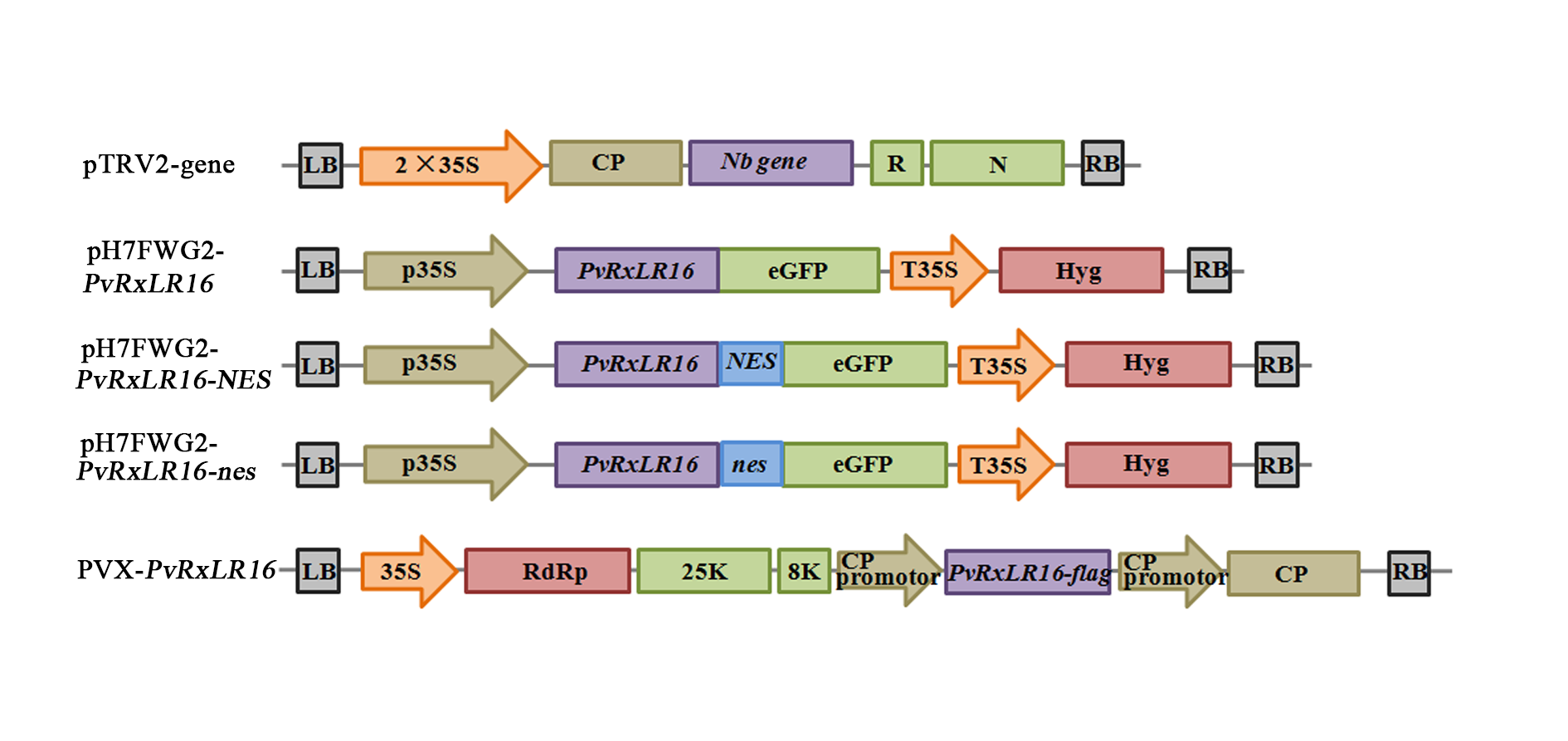

Supplement: Supplementary file 2 — Schematic diagrams of constructs used in this study. (TIFF 1018 kb) [file 12870_2017_1016_MOESM2_ESM.tif]

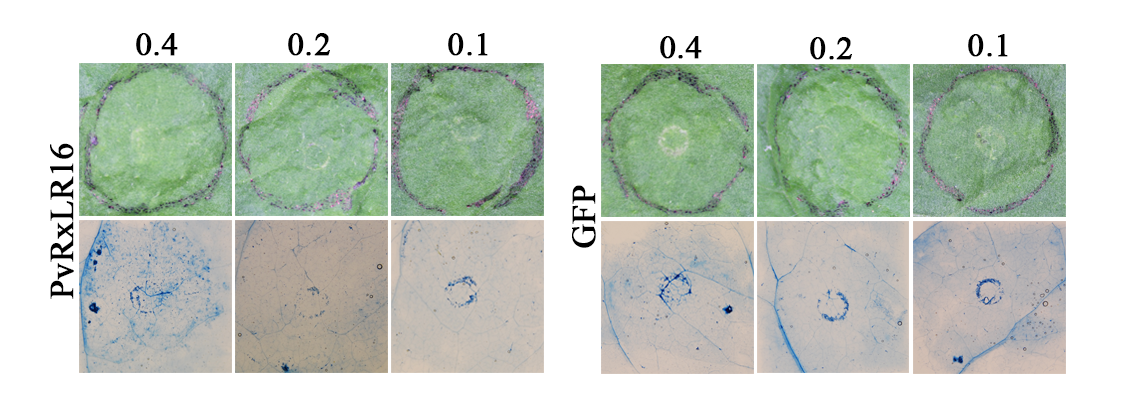

Supplement: Supplementary file 4 — Transient expression of PvRxLR16 and GFP in N. benthamiana. Representative N. benthamiana leaves infiltrated with various concentrations (OD600 = 0.4, 0.2, 0.1) of Agrobacterium suspension containing the PVX-PvRxLR16 or PVX-GFP. Upper pictures, directly photographed 2.5 d post-infiltration. Lower pictures, photographed 2.5 d post-infiltration after staining with trypan blue. (TIFF 1612 kb) [file 12870_2017_1016_MOESM4_ESM.tif]
