# Supplementary material for: A candidate RxLR effector from Plasmopara viticola can elicit immune responses in Nicotiana benthamiana
Source: BMC Plant Biol. 2017 Apr 14;17:75. doi: 10.1186/s12870-017-1016-4 (PMC5391559; doi:10.1186/s12870-017-1016-4)
Supplement: Supplementary file 3 — Genbank accession numbers of genes in N. benthamiana and PvRxLR effectors used in this study. (DOCX 14 kb) [file 12870_2017_1016_MOESM3_ESM.docx]

**Additional file 3: Table S2. Genbank accession numbers of genes in *N. benthamiana* and PvRxLR effectors used in this study**

| Gene | Accession number |
| --- | --- |
| *PvRxLR16* | KX010952 |
| *PvRxLR25* | KX010956 |
| *PvRxLR1* | KX010946 |
| *PvRxLR10* | KX010950 |
| *PvRxLR30* | KX010960 |
| *MAPKKKα* | AY500155.1 |
| *MEK2* | AB360636.1 |
| *SIPK* | AB098730 |
| *MEK1* | AB360635.1 |
| *NTF6* | AB360634.1 |
| *WIPK* | AB098729 |
| *WRKY1* | AY547498.1 |
| *WRKY2* | AY547495.1 |
| *SGT1* | AF516180.1 |
| *HSP90* | GQ845021.1 |
| *RAR1* | AF480487.1 |
| *SERK3* | KJ730242.1 |
